# How the west was won: genetic reconstruction of rapid wolf recolonization into Germany’s anthropogenic landscapes

**DOI:** 10.1038/s41437-021-00429-6

**Published:** 2021-04-12

**Authors:** Anne Jarausch, Verena Harms, Gesa Kluth, Ilka Reinhardt, Carsten Nowak

**Affiliations:** 1 Conservation Genetics Group, Senckenberg Research Institute and Natural History Museum Frankfurt, Gelnhausen, Germany; 2 Department of Biological Sciences, Johann Wolfgang Goethe-University, Frankfurt am Main, Germany; 3 LUPUS – German Institute for Wolf Monitoring and Research, Spreetal, Germany; 4 Present Address: Landesamt für Umwelt Brandenburg, Potsdam, Germany

**Keywords:** Conservation biology, Population genetics

## Abstract

Following massive persecution and eradication, strict legal protection facilitated a successful reestablishment of wolf packs in Germany, which has been ongoing since 2000. Here, we describe this recolonization process by mitochondrial DNA control-region sequencing, microsatellite genotyping and sex identification based on 1341 mostly non-invasively collected samples. We reconstructed the genealogy of German wolf packs between 2005 and 2015 to provide information on trends in genetic diversity, dispersal patterns and pack dynamics during the early expansion process. Our results indicate signs of a founder effect at the start of the recolonization. Genetic diversity in German wolves is moderate compared to other European wolf populations. Although dispersal among packs is male-biased in the sense that females are more philopatric, dispersal distances are similar between males and females once only dispersers are accounted for. Breeding with close relatives is regular and none of the six male wolves originating from the Italian/Alpine population reproduced. However, moderate genetic diversity and inbreeding levels of the recolonizing population are preserved by high sociality, dispersal among packs and several immigration events. Our results demonstrate an ongoing, rapid and natural wolf population expansion in an intensively used cultural landscape in Central Europe.

## Introduction

Wilderness areas are rapidly declining across the planet, while available habitats and population numbers of large mammals shrink globally (Di Marco et al. 2014; Watson et al. 2016). Large carnivores, for instance, are globally threatened due to their lethal persecution by humans in reaction to livestock predation as well as the reduction of habitats and prey availability (Ripple et al. 2014). In the current anthropogenic age, one important way to protect large animals, including large carnivores, may be to foster human–wildlife coexistence within the same landscapes. Ideally, such a strategy requires mutual coadaptation (Carter and Linnell 2016), involving effective human–wildlife conflict management (van Eeden et al. 2018).

Interestingly, the global decline in large animal populations is not ubiquitous across all regions and species. In Europe, for instance, several large carnivore populations (brown bear *Ursus arctos*, grey wolf *Canis lupus*, Eurasian lynx *Lynx lynx* and wolverine *Gulo gulo*) continuously grow due to effective conservation measures and socioeconomic changes (Chapron et al. 2014). Although the rewilding in Europe with large mammal species has raised considerable public and scientific interest and may serve as a case study on the potential for human-large carnivore coexistence in human-dominated landscapes, detailed knowledge about the patterns of range and population expansion into human-dominated landscapes is still limited. In this regard, the grey wolf *Canis lupus* Linæus, 1758 represents a particularly interesting study subject. Wolves are currently recolonizing their historic ranges within several human-dominated landscapes in Western and Central Europe (Chapron et al. 2014).

In Germany, for instance, the first wolf pack was confirmed in 2000 in the Eastern part of the country after more than 150 years without resident wolves (Ansorge et al. 2006). Since then, the population expanded, and is now recognized as the Central European population (Chapron et al. 2014), spreading across mainly Northern Germany (Reinhardt et al. 2019) and Western Poland (Nowak and Mysłajek 2016; Szewczyk et al. 2019), with single individuals dispersing to Denmark (Andersen et al. 2015). Genetic comparisons suggest that the Central European population likely derived from long-distance dispersers from the Baltic wolf population in North-Eastern Poland (Czarnomska et al. 2013). Distant areas are recolonized by wolves due to their high dispersal ability with jump-dispersal events of over 300 km (Kojola et al. 2006; Wabakken et al. 2007; Ciucci et al. 2009; Ražen et al. 2016). Despite their potential of long-distance dispersal, a large proportion of dispersing individuals settle within 100 km from their natal packs (Kojola et al. 2006; Caniglia et al. 2014).

While there is considerable knowledge about wolf dispersal in natural or semi-natural areas (reviewed in Mech and Boitani 2003), few studies have documented the mechanisms of range extension and population establishment in more densely populated, anthropogenic landscapes. Few detailed multigenerational pedigrees documenting the expansion of wolf populations have so far been generated and are mostly derived from sparsely human-populated northern regions such as Scandinavia and Yellowstone National Park (Liberg et al. 2005; vonHoldt et al. 2008; Granroth-Wilding et al. 2017), or in the Apennine Mountains in Northern Italy (Caniglia et al. 2014). In this study, we summarize the results of intense genetic wolf monitoring during the initial 15 years of wolf recolonization in Germany to reconstruct patterns of dispersal and population expansion into an intensively human-dominated landscape. We hypothesized that basic patterns of range expansion in wolves would be similar in a human-dominated landscape as in more natural habitats. Considering the large distance (>400 km) to the source population in Eastern Poland as well as the dispersal patterns known for wolves in other areas, including Poland (Nowak and Mysłajek 2016; Szewczyk et al. 2019), we predicted that (i) we would find an initial founder effect during the early colonization phase and that (ii) recolonization would follow a similar process to that found in other areas (Mech and Boitani 2003). This process typically starts with an initial pack from which the offspring disperse into neighbouring areas. The local dispersers of the original pack then usually reproduce with newly immigrated individuals. We also expected that (iii) gene flow and dispersal among packs would be predominantly male-biased (vonHoldt et al. 2008; Caniglia et al. 2014). Moreover, we supposed that (iv) genetic diversity would be lower and inbreeding levels would be higher of the recolonizing population compared to larger, persistent European wolf populations (Hindrikson et al. 2017). Based on the genealogical data, we survey trends in genetic diversity, inbreeding and population structure.

## Materials and methods

### Study area and sample collection

Within the European Union (EU), where Germany is a member state, the wolf is listed in Annex II and IV under the conservation legislation of the EU Habitats Directive (Council Directive 92/43/EEC), with the overall goal of reaching the ‘Favourable Conservation Status, FCS’ (Article 2, Council Directive 92/43/EEC). Included in Article 2, the conservation status of the wolf as priority species needs to be monitored by the member states (Article 11). The wolf population in Germany has been monitored since 2001. The main monitoring methods used in Germany are presence sign surveys in combination with camera trapping and genetic analyses (Kaczensky et al. 2009; Reinhardt et al. 2015). The major objectives of the German wolf monitoring are the annual assessment of the area of occurrence and the population size given as the minimum number of packs (including reproductions), scent-marking pairs and territorial single wolves.

In Germany, all wolf monitoring activities are coordinated and conducted by the 16 federal states, following the German monitoring standards for large carnivores (Reinhardt et al. 2015), where genetic analyses constitute a key part. Searching for genetic samples such as scats or urine traces is usually conducted on regional scales by a network of trained persons coordinated by the responsible State Environmental Agencies. Due to the decentralized local responsibilities, monitoring intensity and strategies for sample collection vary in space and time. However, genetic samples are regularly collected in all federal states with occasional or regular wolf presence. As the monitoring activities during the initial phase of wolf recolonization were particularly intense, we assume that all packs were identified at least until 2013.

In this study, scats made up most of the genetic material used for monitoring purposes and were generally collected all year-round during presence sign surveys. Other frequently collected sample types were hair (e.g. from day beds), urine and blood stains during the pre-oestrus period collected while snow tracking. Saliva traces were collected from livestock and wild ungulate kills. Tissue samples were collected from wolf carcasses and a tooth from one set of skeletal remains. Blood, hair and saliva samples were collected from some wolf carcasses in addition to tissue samples. Blood samples were collected from injured wolves. Blood, hair or saliva samples were collected from wolves captured for radio collaring.

**Fig. 1:**
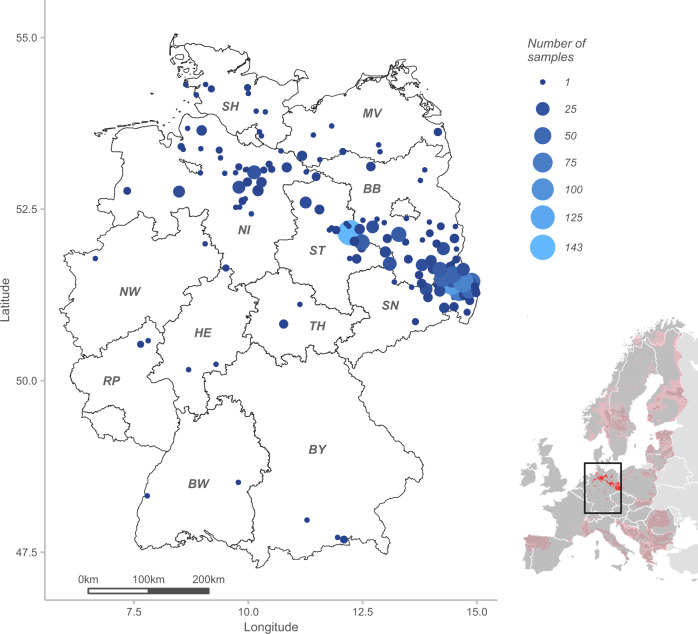
Wolf study area and sampling localities cover the whole of Germany and are divided into the 16 federal states (black lines). Shown are the number of successfully genotyped samples collected between 2003 and April 2016 (blue circles) in 12 federal states (labelled with grey initials, see Tables S1–S3). The smaller map shows the wolf distribution across Europe in 2011 with permanent occurrence (dark pink) and sporadic occurrence (pale pink) according to Chapron et al. (2014) and the confirmed wolf occurrence in Germany for 2015 (red) according to the Dokumentations- und Beratungsstelle des Bundes zum Thema Wolf (2017).

**Fig. 2:**
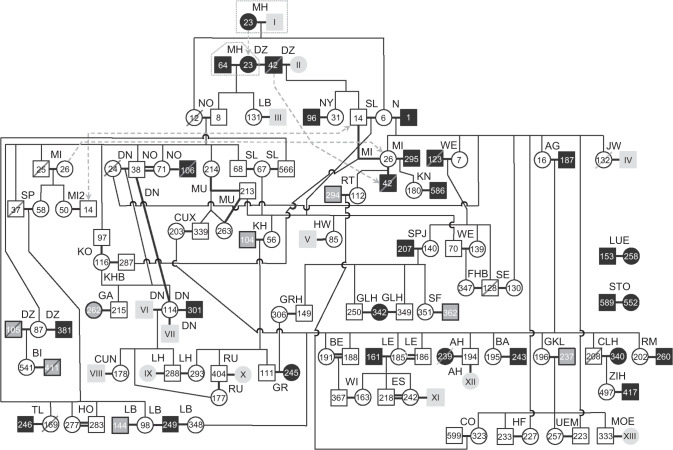
Pedigree of reproducing German wolves in the monitoring period 2005–2015 reconstructed from a combination of microsatellite, mtDNA and field data. Bold black lines symbolize successful reproductions between female (circles) and male (squares) wolves, double bold black lines highlight breeding of full-siblings, while thin black lines indicate parent–offspring relationships. Four individuals are illustrated twice (arrow with dashed grey line). Individuals with known source pack carrying haplotype HW01 (unfilled), individuals with unknown source pack carrying HW01 (filled with black) and individuals with unknown source pack carrying haplotype HW02 (filled with dark grey). Individuals found dead are crossed out. Individuals with missing genotype are indicated in light grey with Roman numerals. Breeding pairs in which both breeders were not genotyped are not depicted. Framed with a grey dotted line are the first (GW023f and I from 2000 to 2001) and the second breeding pair (GW023f and GW064m from 2002 to 2004) in the Muskauer Heide (MH), which are not included in the further analyses on trends of genetic diversity and inbreeding of breeding pairs until 2015 (see ‘Materials and Methods’).

Within the first years of German wolf monitoring, initial genetic analyses were performed at the Institute of Nature Conservation, Polish Academy of Sciences, in Krakow, Poland (Reinhardt and Kluth 2007). In 2009, all federal states of Germany agreed to use the Senckenberg Research Institute as the central laboratory for genetic wolf analyses to guarantee the generation of harmonized data among the federal states. In this study, we used wolf samples that were collected throughout Germany (47°16′–55°03′ N and 5°52′–15°02′ E) between January 2003 and April 2016 in the frame of the federal-state-based local long-term monitoring activities (Fig. 1). We started from 2005 with the third and fourth breeding pair (GW006f and GW001m in N; GW012f and GW008m in NO, see Tables S1 and S2 for details on individual genotype ID and wolf territories, respectively) to describe the trends of genetic diversity and inbreeding of the breeding pairs until 2015; between 2000 and 2004 only two successive breeding pairs and a hybridization event occurred. The first wolf reproduction was documented in the Muskauer Heide (MH), Saxony, in 2000 (Ansorge et al. 2006). Monitoring and genetic data suggest that the female GW023f and the male wolf ‘I’ with missing genotype formed the first breeding pair (Fig. 2), which reproduced in 2000 and 2001. From 2002 to 2004, GW023f reproduced with another male GW064m in MH. Furthermore, a hybridization event occurred in 2003 (Reinhardt and Kluth 2007). Microsatellite analysis revealed that the female GW006f mated with a domestic dog adjacent to her natal territory. In winter 2003/2004, four hybrid pups were still alive. Two of them were caught and brought into an enclosure, while the other two hybrids disappeared. The F1-hybridisation event was confirmed by further analyses based on 93 single-nucleotide polymorphisms (Harmoinen et al. 2020).

### DNA extraction and genotyping

DNA from faecal samples was extracted using the QIAamp DNA Stool Mini Kit (Qiagen, Hilden, Germany). For DNA extraction, mucus was scraped off from the surface of the faeces with forceps. DNA extraction from urine and blood (oestrus) samples was performed following Hausknecht et al. (2007) with slight modifications. In short, DNA and cellular remains were precipitated from ~15 ml of urine–snow mixture by addition of 1.5 ml of sodium acetate (3 M; pH 5.0) and 33 ml of ethanol (96%). After overnight incubation at −20 °C, DNA was pelleted by cold centrifugation (3600 rpm, 60 min and 4 °C). The DNA extraction was then performed using the QIAamp DNA Stool Mini Kit (Qiagen, Hilden, Germany). For tissue samples, DNA from ~25 mg of tissue was extracted using the DNeasy^®^ Blood & Tissue Kit (Qiagen, Hilden, Germany). DNA from saliva, saliva traces from kills, blood, hair samples and one tooth was extracted using the QIAamp DNA Investigator Kit (Qiagen, Hilden, Germany). In each case of extraction, manufacturers’ protocols were followed.

Mitochondrial DNA haplotypes were determined by sequencing a short stretch of the mitochondrial control region (250 or 390 bp). Saliva samples and one tooth sample were sequenced using the primers WDloopL and WDloopH254 (Caniglia et al. 2013). PCRs were performed in 10-μl volumes containing 2.2-μl molecular grade water, 5-μl 1 × SensiFAST SYBR^®^ No-ROX (Bioline GmbH, Luckenwalde, Germany), 0.4 μM of each primer and 2-μl DNA extract. The PCR protocol started with initial denaturation at 95 °C (3 min), following 40 cycles of 95 °C (5 s) and 60 °C (30 s). The laboratory procedures and protocols for mtDNA sequencing of scat, hair, urine, blood and tissue samples as well as purification of PCR products have been described in Lesniak et al. (2017). Sequencing was carried out on an ABI 3730 DNA Analyzer (Life Technologies, Carlsbad, California, USA). Sequences were analysed in GENEIOUS ver. 7.1.9 (Biomatters Ltd, Auckland, New Zealand) and compared to sequences deposited in the NCBI database as well as to our internal reference haplotype database.

Autosomal microsatellite data were obtained as part of the regular genetic wolf monitoring performed in our laboratory to assess relatedness and origin of the wolves. We used 13 variable unlinked microsatellites along with two sex markers, DBX6 and DBY7 (Seddon 2005). Markers CPH5 (Fredholm and Winterø 1995), FH2001, FH2010, FH2017, FH2054, FH2087L, FH2088, FH2096, FH2137, FH2140, FH2161 (Francisco et al. 1996), vWF (Shibuya et al. 1994), PEZ17 (Neff et al. 1999) and the two sex markers were amplified in three multiplex PCRs. Each microsatellite PCR of 10 µl consisted of 2X HotStarTaq Master Mix (Qiagen, Hilden, Germany), 0.2 µM of each primer, 2-ng BSA and 3.6 µl of DNA template. PCRs were performed in a T1 plus Thermocycler (Analytik Jena AG, Jena, Germany). Initial denaturation was set to 95 °C for 15 min, followed by four cycles of 94 °C for 30 s, 60 °C for 90 s and 72 °C for 60 s; five cycles of 94 °C for 30 s, 58 °C for 90 s and 72 °C for 60 s; five cycles of 94 °C for 30 s, 54 °C for 90 s and 72 °C for 60 s; and twenty-five cycles of 94 °C for 30 s, 50 °C for 90 s and 72 °C for 60 s with an final extension at 72 °C for 30 min. PCR products were diluted 1:5 prior to fragment length analysis performed on an ABI 3730 DNA Analyzer (Applied Biosystems, Foster City, CA, USA). Fragment sizes were determined using the software GENEMARKER ver. 1.90 (Softgenetics LLC, State College, Pennsylvania, USA) by comparison to the GeneScan™600 LIZ^®^ size standard (Applied Biosystems, Foster City, CA, USA). A multiple-tube approach was applied including at least four and, for some samples, up to eight or twelve replicates per non-invasive sample. Consensus genotypes with a minimum of ten loci were accepted with ≥2 PCR amplifications of a heterozygote and ≥3 PCR amplifications of a homozygote locus. Some selected samples were included despite a lower amplification success rate. For these individuals, fragmentary genotypes were verified and completed by comparison with the known genotypes of the breeding partner and pups of that breeding pair. One mixed DNA sample (saliva traces from killed prey) was also included, as it contained the genotypes of the two breeders in that territory.

### Data analyses

To identify individual genotypes, the R package DNAtools (Tvedebrink et al. 2012; Curran and Tvedebrink 2013) in the R programming language (R Core Team 2017) was used. Descriptive statistics of microsatellite loci and probabilities of identity were performed with GenAlEx ver. 6.5 (Peakall and Smouse 2006, 2012). The mean number of different alleles per microsatellite locus (Na), number of effective alleles (Ne), observed heterozygosity (Ho) and unbiased expected heterozygosity (He) were calculated, as well as the probability of identity (PID) and probability of identity between siblings (PIDsib) (Waits et al. 2001). As the number of distinct alleles depend on sample size, we additionally calculated allelic richness (Ar) using the rarefaction approach as implemented in ADZE ver. 1.0 (Szpiech et al. 2008). Tests for scoring errors caused by stutter peaks, large allelic dropout and the presence of null alleles were performed in MICRO-CHECKER ver. 2.2.3 (van Oosterhout et al. 2004). CERVUS ver. 3.0.7 (Kalinowski et al. 2007) was used to calculate the polymorphism information content (PIC) and to create input files for the software GENEPOP ver. 4.2 (Raymond and Rousset 1995; Rousset 2008) to conduct Hardy–Weinberg Equilibrium (HWE) testing; dememorization number = 5000; number of batches = 1000; number of iterations per batch = 5000. To detect sex-biased dispersal based on microsatellite markers, the powerful mean of the corrected assignment index (mAIc) tests (Goudet et al. 2002) was conducted using the R package hierfstat ver. 0.04–22 (Goudet 2005).

### Pedigree reconstruction, genetic diversity and inbreeding

For pack and pedigree reconstruction, genetic data were combined with additional information recorded in the frame of the national wolf monitoring, including spatio-temporal data on, for example, wolf occurrence, territories, social status of individuals or evidence of reproductions. As wolves live in families (Mech and Boitani 2003), the assignment of individuals to the respective packs allows for reconstructing a continuous pedigree across several generations. A ‘breeding pair’ consists of two reproducing adult wolves, while a ‘pack’ is the wolf family comprising the breeding animals and their offspring. The ‘territory’ is the home range inhabited and defended by the pack (Mech and Boitani 2003). From all collected DNA samples, the scent-marking individuals and possible pups in the respective territories were identified and separated from other breeding pairs and pups in adjacent territories. Individuals that were identified outside of the distribution range, at the range edges or in areas lacking active monitoring were checked against the known packs and pairs for a possible assignment as offspring.

Various computer programmes are available for inferring kinship and pedigree reconstruction (Jones et al. 2010; Walling et al. 2010). However, software-supported parentage assignments frequently contain mismatches (Walling et al. 2010). Thus, for accurate and robust pedigree reconstruction, we combined manual analyses of genetic and monitoring data additionally supported by parentage assignments of the program COLONY ver. 2.0.6.4 (Jones and Wang 2010). Male and female breeding systems were set to ‘polygamous’ and the inbreeding model was selected. Pack numbers and territories determined in the German monitoring were obtained from the public wolf database (DBBW 2017, https://dbb-wolf.de/). As wolf pups are born at the end of April/beginning of May, a wolf monitoring year starts at the first of May and ends at the end of April in the following year. Here, we mention the year in which the monitoring year starts (e.g. 2005 for the monitoring year 2005/2006).

Based on the pedigree data, the mean number of alleles (Na), allelic richness (Ar), observed heterozygosity (Ho) and unbiased expected heterozygosity (He) were calculated for the different years based on the genotypes of the inferred breeding pairs to evaluate the genetic diversity. Pedigree-based inbreeding coefficients (Fp) for the offspring from breeding pairs were calculated with the inbreeding function in the R package GeneticsPed ver. 1.40.0 (Gorjanc and Henderson 2007) using the method ‘meuwissen’ (Meuwissen and Luo 1992). For calculating pedigree-based inbreeding coefficients, individuals with an unknown source pack were considered as unrelated founders or immigrants. We fitted an exponential growth model to the yearly genotyped breeding pairs as well as to the yearly numbers of breeding pairs inferred by the overall wolf monitoring activities and calculated the annual growth of reproductive units from the model using the statistical programming language R (R Core Team 2017). For the detection of trends in the genetic diversity parameters described above, we performed the Mann–Kendall test (Libiseller and Grimvall 2002) and calculated Sen’s Slope (Sen 1968) using the R package TREND (Pohlert 2018).

To better understand the recolonization process, we defined three core areas (CORE1, CORE2, CORE3, see Fig. S1) based on the spatio-temporal data of all territories of the genotyped breeding pairs identified between 2005 and 2015. We constructed spatio-temporal networks of territories, starting with the first three territories of wolf packs, which established further north–west from a considerable distance to already existing territories: Neustadt (N) in 2005 for core area 1, Altengrabow (AG) in 2009 for core area 2 and Munster (MU) in 2012 for core area 3. The remaining territories were assigned to the respective core area according to the smallest spatial and temporal distance to one of the three predefined core areas. We created a minimum convex polygon for each core area based on the constructed networks of our territory data. The territory of the UEM pack was not included as the geographical position and time of pack formation did not allow for a clear assignment to a distinct core area.

In order to gain insights into the genetic diversity of the reproducing individuals in the three core areas, we calculated the mean number of alleles (Na), number of effective alleles (Ne), allelic richness (Ar), observed heterozygosity (Ho) and unbiased expected heterozygosity (He) for the year 2015. Furthermore, we used the fast maximum-likelihood clustering method ‘snapclust’ (Beugin et al. 2018) in the R package ADEGENET ver. 2.1.1 (Jombart 2008) to reveal spatial genetic patterns in the expanding wolf population. The snapclust.choose.k function was used to identify the optimal number of clusters (*K*) based on the Bayesian information criterion (Schwarz 1978) for the reproducing individuals in the year 2015.

Overall comparison of dispersal distances of breeding females and males was conducted with the Wilcoxon rank-sum test and observed to expected distribution of the sex ratio was compared with the chi-square test in R. Linear distances between the centres of the natal territory to the territory of the first reproduction were calculated with the R package GEOSPHERE ver. 1.5–7 (Hijmans et al. 2017) using the ‘Vincenty Ellipsoid’ method. For the analysis of dispersal distances, we tested two subsets, (i) including breeding individuals that stayed and reproduced in their natal territory, and (ii) including only breeding individuals that left their natal territory. For breeders that reproduced in multiple years with the same or different breeding partners, however, only the distance between the first reproduction and the natal territory was considered.

## Results

We successfully genotyped 1341 samples collected between 2002 and 2015 across Germany, including 872 scats, 126 saliva traces from killed prey, 108 tissue and 108 urine samples, 78 hair samples, 34 blood samples, eight oestrus blood stains, six saliva samples collected from carcasses or from wolves captured alive and one tooth. Overall, we identified 524 individuals in the analysed samples (Table S1). Wolf individuals were genotyped from 1 to 18 times, and 43.3% of the genotypes were identified only once. Individuals were sampled 2.6 times on average. No evidence of frequency distortion through large allele drop-outs or stutter peaks and no null alleles were identified for the microsatellite loci, except for locus PEZ17 where null alleles may be present. When testing all 524 genotyped individuals that were identified between 2002 and 2015, six of the 13 loci deviated significantly from HWE (*p* < 0.05). Testing of the breeder’s datasets for each year from 2005 to 2015 revealed that <6% of the loci across all eleven subsets deviated significantly from HWE (*p* < 0.05). By comparing inheritance patterns with field data, 7 of 13 loci exhibited consistent Mendelian patterns of inheritance. In 17 of the 431 offspring genotypes (3.9%), one allele at one of the six loci (CPH5, FH2137, FH2161, FH2054, FH2088 or PEZ17) did not match between parents and offspring. As the rate was low, all 13 loci were used for subsequent analyses. For all 524 identified individuals, the mean number of alleles was 6.62, observed heterozygosity was 0.574 and expected heterozygosity was 0.573. The polymorphic information content for the set of 13 loci was high (PIC = 0.526). The probability of identity (PID) was 2.8 × 10^−09^ and the probability of identity between siblings (PIDsib) was 1.85 × 10^−04^, indicating that the presence of individuals sharing the same genotype by chance was very unlikely within the population.

### Relatedness among breeding individuals

**Fig. 3:**
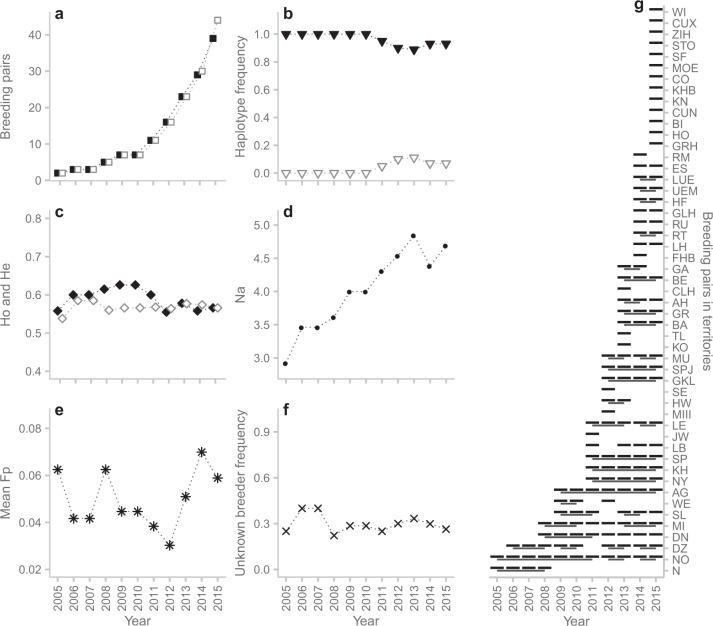
Trends in genetic diversity and exponential increase of breeding pairs in Germany between 2005 and 2015. **a** Yearly numbers of genotyped breeding pairs (black squares) compared to the numbers of all confirmed breeding pairs (grey unfilled squares). **b** Yearly haplotype frequency (HW01 [black triangles] and HW02 [grey unfilled triangles]) of breeders. **c** Mean yearly observed (Ho; black diamonds), unbiased expected (He; grey unfilled diamonds) heterozygosity levels and **d** mean number of alleles (Na; black dots) of the breeding individuals. **e** Yearly average pedigree-based inbreeding coefficients (mean Fp) of the offspring from the breeding pairs (black asterisks). **f** Yearly frequency of breeders with unknown source packs (black crosses). **g** Genotyped breeding pairs in the different territories. For breeding pairs that persisted over several years, lines connect the respective years.

Pack and pedigree analysis based on genetic and field data allowed us to determine the relatedness among the identified individuals. This was done for 76 different breeding pairs in 51 territories with a total of 145 litters between 2005 and 2015 (including 648 pups confirmed by the German wolf monitoring, 431 of which were genotyped) (Tables 1, S2 and Figs. 2, S2). Compared to the total of 151 breeding pairs confirmed by the German wolf monitoring, 96.03% of the breeding pairs in the population were genotyped until 2015 (Table S4). Thus, we were able to reconstruct a near-complete pedigree. Seventy-nine (69.9%; 31 males, 48 females) of the 113 genotyped breeders were born in a German pack, while 34 breeders (30.1%; 26 males, 8 females) could not be assigned to a genetically known pack. As these individuals showed no first-order relationship to known German packs, they were considered likely to be immigrants. Various breeding pairs persisted over several years, while in some territories multiple breeder turnovers occurred with the result that some individuals reproduced with different breeding partners (Table 1 and Figs. 2, 3g). Several breeding pairs between related wolves were identified, including five full-sibling breeding pairs and two parent–offspring breeding pairs (see Fig. 2 and the following paragraph).

**Table 1:** Reproducing breeding pairs identified between 2005 and 2015 in Germany (*N* = 76), with initial of the territory (T, see Table S2), breeding male ID (BM) and female ID (BF) with initials of their natal pack (unk. = natal pack unknown), pedigree-based inbreeding coefficient (Fp) of the offspring of the respective breeding pair and the known breeding years (Years).

No.	T	BM	BF	Fp	Years	No.	T	BM	BF	Fp	Years
1	AG	GW187m (unk.)	GW016f (N)	0	2009–15	39	LB	GW249m (unk.)	GW098f (SP)	0	2014
2	AH	GW194m (AG)	GW239f (unk.)	0	2013–14	40	LB	GW249m (unk.)	GW348f (AG)	0	2015
3	AH	GW194m (AG)	XII (unk.)	0	2015	41	LE	GW161m (unk.)	GW185f (AG)	0	2011–13
4	BA	GW243m (unk.)	GW195f (AG)	0	2013–15	42	LE	GW186m (AG)	GW185f (AG)	0.25	2014–15
5	BE	GW188m (AG)	GW191f (AG)	0.25	2013–15	43	LH	GW288m (DN)	GW293f (KH)	0.027	2014
6	BI	GW411m (unk.)	GW541f (DZ)	0	2015	44	LH	GW288m (DN)	IX (unk.)	0	2015
7	CLH	GW208m (AG)	GW340f (unk.)	0	2013	45	LUE	GW153m (unk.)	GW258f (unk.)	0	2014–15
8	CO	GW599m (RT)	GW323f (GKL)	0.016	2015	46	MI	GW025m (NO)	GW026f (N)	0.094	2008–10
9	CUN	VIII (unk.)	GW178f (DN)	0	2015	47	MI	GW042m (unk.)	GW026f (N)	0	2011
10	CUX	GW339m (MU)	GW203f (AG)	0.055	2015	48	MI	GW295m (unk.)	GW026f (N)	0	2012
11	DN	GW038m (NO)	GW024f (N)	0.094	2008–11	49	MI	GW014m (DZ)	GW026f (N)	0	2013–15
12	DN	VI (unk.)	GW114f (DN)	0	2012	50	MI2	GW014m (DZ)	GW050f (MI)	0	2012
13	DN	GW038m (NO)	GW114f (DN)	0.328	2013	51	MOE	GW333m (GKL)	XIII (unk.)	0	2015
14	DN	GW301m (unk.)	GW114f (DN)	0	2014	52	MU	GW213m (SL)	GW214f (NO)	0.094	2012–13
15	DN	VII (unk.)	GW114f (DN)	0	2015	53	MU	GW213m (SL)	GW263f (MU)	0.297	2014–15
16	DZ	GW042m (unk.)	II (unk.)	0	2006–08	54	N	GW001m (unk.)	GW006f (MH)	0	2005–08
17	DZ	GW042m (unk.)	GW023f (unk.)	0	2009–10	55	NO	GW008m (MH)	GW012f (MH)	0.125	2005–11
18	DZ	GW105m (unk.)	GW087f (NO)	0	2012–13	56	NO	GW106m (unk.)	GW071f (NO)	0	2012–13
19	DZ	GW381m (unk.)	GW087f (NO)	0	2014–15	57	NO	GW038m (NO)	GW071f (NO)	0.313	2014–15
20	ES	GW218m (LE)	GW242f (LE)	0.25	2014	58	NY	GW096m (unk.)	GW031f (DZ)	0	2011–15
21	ES	XI (unk.)	GW242f (LE)	0	2015	59	RM	GW260m (unk.)	GW202f (AG)	0	2014
22	FHB	GW128m (WE)	GW347f (WE)	0.156	2014	60	RT	GW294m (unk.)	GW112f (MI)	0	2014–15
23	GA	GW215m (DN)	GW262f (unk.)	0	2013–14	61	RU	GW404m (KH)	X (unk.)	0	2014
24	GKL	GW237m (unk.)	GW196f (AG)	0	2012–15	62	RU	GW404m (KH)	GW177f (DN)	0.027	2015
25	GLH	GW250m (SPJ)	GW342f (unk.)	0	2014	63	SE	GW128m (WE)	GW130f (N)	0.125	2012
26	GLH	GW349m (SPJ)	GW342f (unk.)	0	2015	64	SF	GW362m (unk.)	GW351f (SPJ)	0	2015
27	GR	GW111m (KH)	GW245f (unk.)	0	2013–15	65	SL	GW014m (DZ)	GW006f (MH)	0	2009–11
28	GRH	GW149m (SPJ)	GW306f (GR)	0.008	2015	66	SL	GW068m (NO)	GW067f (SL)	0.094	2013–14
29	HF	GW233m (GKL)	GW227f (AG)	0.125	2014–15	67	SL	GW566m (SP)	GW067f (SL)	0.102	2015
30	HO	GW283m (SP)	GW277f (SP)	0.379	2015	68	SP	GW037m (NO)	GW058f (MI)	0.203	2011–15
31	HW	V (unk.)	GW085f (SL)	0	2012–13	69	SPJ	GW207m (unk.)	GW140f (WE)	0	2012–15
32	JW	IV (unk.)	GW132f (N)	0	2011	70	STO	GW589m (unk.)	GW552f (unk.)	0	2015
33	KH	GW104m (unk.)	GW056f (SL)	0	2011–15	71	TL	GW246m (unk.)	GW169f (SP)	0	2013
34	KHB	GW287m (MI)	GW116f (DN)	0.086	2015	72	UEM	GW223m (N)	GW257f (GKL)	0.063	2014–15
35	KN	GW586m (unk.)	GW180f (MI)	0	2015	73	WE	GW123m (unk.)	GW007f (N)	0	2009–10
36	KO	GW097m (NO)	GW116f (DN)	0.203	2013	74	WE	GW070m (SL)	GW139f (WE)	0.063	2012
37	LB	III (unk.)	GW131f (MH)	0	2011	75	WI	GW367m (BE)	GW163f (LE)	0.125	2015
38	LB	GW144m (unk.)	GW098f (SP)	0	2013	76	ZIH	GW417m (unk.)	GW497f (CLH)	0	2015

Breeding individuals with missing genotype are indicated with Roman numerals.

### Trends in genetic diversity and inbreeding

The German wolf population re-expanded since the first reproduction in 2000 with an exponential increase of 30.5% for all confirmed breeding pairs and annual growth rates of 29.8% for genotyped breeding pairs (Fig. 3a and Table S4). In 2005, two genotyped and confirmed breeding pairs reproduced, while in 2015, reproduction was found for 39 genotyped out of 44 confirmed breeding pairs. We found four wolf mtDNA haplotypes among all 524 identified individuals: HW01, HW02, HW03 and HW22, nomenclature adapted to Pilot et al. (2010). HW01, HW02 and HW03 occur widely in North-Eastern and Central Europe, while haplotype HW22 is largely predominant private for the Italian and Alpine wolf populations (Pilot et al. 2010). HW22 can be regarded as reliable indication of Italian or Alpine population origin, as other haplotypes are rarely found in these two populations (Dufresnes et al. 2018). The reproducing individuals only carried haplotypes HW01 and HW02 (Figs. 2 and 3b). One hundred and five breeders carried HW01, while only eight breeders, seven males and one female, carried haplotype HW02. The haplotype frequencies declined (HW01) and increased (HW02) significantly over time (*p* = 0.01, Sen’s Slope = ±0.009) (Figs. 2, 3b and Table S4). HW01 was detected with a much higher frequency (89–100%) in the breeding individuals than HW02 (5–11%), which first appeared in 2011. HW03 was found in one individual by a single scat sample collected 2014 in North-Eastern Germany. Six male wolves carried haplotype HW22 (four were found dead and two were identified once at killed ungulates).

Mean yearly observed heterozygosity across microsatellite loci ranged between 0.56 and 0.63, while expected heterozygosity ranged between 0.54 and 0.59. No significant trends in heterozygosity were detected over time (Fig. 3c and Table S4). The mean number of alleles in the breeding individuals increased significantly (*p* < 0.001, Sen’s Slope = 0.179) from 2.9 in 2005 to 4.8 in 2013 (Fig. 3d and Table S4), while the mean allelic richness (Ar) ranged between 1.54 and 1.58 (no significant trend). The yearly average inbreeding coefficients of litters from breeding pairs were low (Mean Fp between 0.03 and 0.07) and no significant trend was detected (Fig. 3e and Table S4). The increase in the mean Fp observed in the last 3 years was associated with nine litters from five full-sibling breeding events. The highest pedigree-based inbreeding coefficient (Fp = 0.379) was found for the litter of the breeding pair in HO in 2015. From 2013 to 2015, three litters originated from daughter–father breeding events where the grandparents were already closely related, resulting in inbreeding coefficients of Fp = 0.297 and 0.328. Overall, 51 of the total 145 litters (35.2%) resulted from breeding with close relatives (Fp = 0.008–0.379), while only 19 litters (13.1%) showed increased inbreeding coefficients Fp = 0.156–0.379 (see Figs. S3 and S4). The yearly frequency of breeders with unknown source pack ranged between 22 and 40% (mean = 29.9%; no significant trend) (Fig. 3f and Table S4). Throughout the study period, the 76 breeding pair bonds lasted on average 1.91 years (between 1 and 7 years, Fig. 3g), albeit some breeding pairs considered in this study persisted beyond the year 2015.

### Breeder dispersal and pack dynamics

**Fig. 4:**
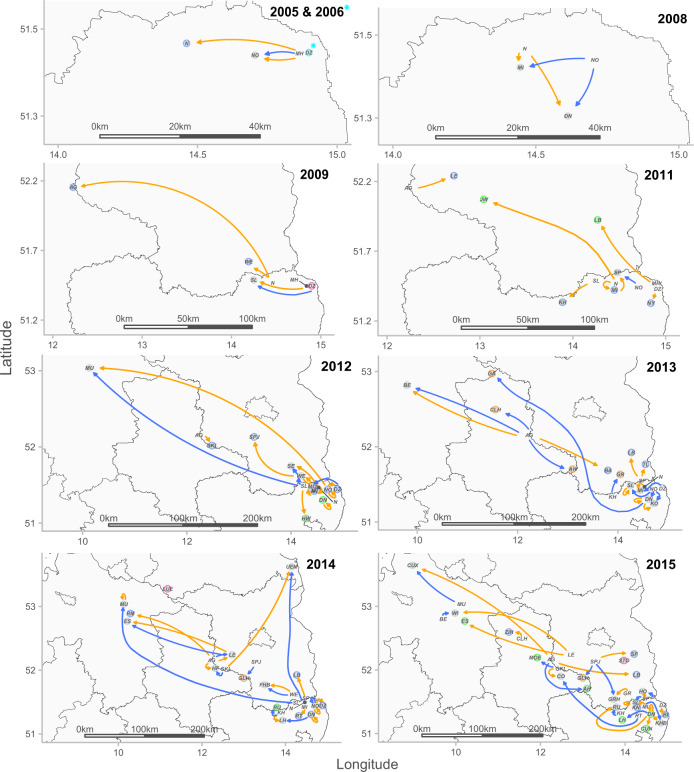
Schematic representation of breeder dispersal and pack dynamics during wolf recolonization in Germany from 2005 to 2015. Shown are wolf territories where a newly established pair reproduced for the first time (grey circles) in the study area (divided into the federal states [grey lines]). Only breeders with known source pack are shown (females [orange arrows] and males [blue arrows]). Breeding partners with unknown source pack are indicated by a blue (male), an orange (female) or a pink (male and female) border around the respective territories, while breeders with missing genotype are indicated by a green border (one parent). The cyan border around DZ indicates the male with unknown source pack and the female with missing genotype. Wolf territories in which genotypes were missing for both breeders are not depicted.

**Fig. 5:**
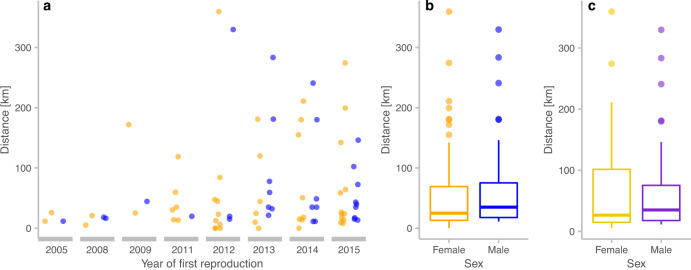
Linear dispersal distances [km] of breeding wolves in Germany for the years 2005 to 2015. **a** Dispersal distances for females (*n* = 48) and males (*n* = 31) that reproduced in the respective years for the first time. **b** Dispersal distances for all females (*n* = 48) and males (*n* = 31) with known natal packs. **c** Dispersal distances for females (*n* = 43) and males (*n* = 31), excluding the five females that stayed and reproduced in their natal territory. No significant differences between dispersal distances of females and males were detected.

In the first years of recolonization (until 2011), 12 out of 13 female breeders originated from German packs, while 5 out of 12 male breeders were born in the study area (Fig. 4). The first reproduction in considerable distance (about 170 km) north–west from the initial core area occurred in 2009, when the female GW016f born in the N territory reproduced with the immigrant male GW187m forming the AG breeding pair. Another successful reproduction occurred more than 300 km north–west from the initial core area in 2012, as the two first-cousins male GW213m and female GW214f originating from SL and NO established the initial MU pack. Consequently, both dispersing males and females became breeders in territories established far from the nearest territory or core area (Figs. 4 and 5). However, female dispersers or female offspring primarily initiated the establishment of breeding pairs in territories adjacent to their source packs (38 females versus 22 males). Mostly dispersing males became new breeding partners in already established packs, while the breeding female remained constant (nine males versus two females). In addition, only female offspring took over breeding positions in their natal territory and formed another breeding pair together with a new male. In 2014, the female offspring GW263f in MU took over the mother’s position of GW214f and reproduced with her father GW213m.

The overall male-biased dispersal was also confirmed by the sex-biased dispersal test, as males had a lower mean of the corrected assignment index (mAIc) than females (*p* < 0.001, mAIc females = 2.131; mAIc males = −2.077). Moreover, we found several cases of serial monogamy where females had between two and four breeding partners (see Table 1 and Fig. 2).

The spatio-temporal expansion process characterized by initial recolonization far from the next source pack and subsequent colonization in the surrounding areas indicated the formation of three core areas (see Fig. S1). We found no substantial differences when comparing microsatellite diversity indices of the reproducing individuals in 2015 among these three areas (see Table S5). Genetic diversity was similar between CORE1, CORE2 and CORE3, with mean observed heterozygosity values ranging between 0.55 and 0.58 and expected heterozygosity values ranging between 0.55 and 0.57. Snapclust analysis indicated that the most likely number of genetic clusters was *K* = 3 including all reproducing individuals for the year 2015 (see Fig. S5).

Linear dispersal distances between the centres of the natal territory to the territory of the first reproduction ranged from 0 to 359.5 km (Fig. 5a–c). Both the shortest and longest dispersal distance for wolves that became successful breeders were recorded for females. The sex ratio of breeders with data on dispersal distance, including individuals that stayed and reproduced in their natal territories, was 48 females (60.8%) versus 31 males (39.2%) and did not deviate from parity (*χ*^2^ = 3.658, df = 1, *p* value = 0.06). Dispersal distances between the reproducing females and males did not differ significantly (Wilcoxon rank-sum test, *W* = 629.5, *p* value = 0.25, Fig. 5b). Mean dispersal distance for females was 62.4 and 71.4 km for males, while the median was 25 km for females and 35 km for males. After excluding the five females that stayed and reproduced in their natal territories, the sex ratio was 43 females (58.1%) versus 31 males (41.9%) (*χ*^2^ = 1.946, df = 1, *p* value = 0.16). Dispersal distances between the reproducing females and males still did not differ significantly (Wilcoxon rank-sum test, *W* = 629.5, *p* value = 0.69, Fig. 5c). Mean dispersal distance for females slightly increased to 69.7 km (median 26.4 km), while dispersal distances for males did not change (see above).

## Discussion

The genetic analysis of various sample types collected over a decade within the framework of the legally required German wolf monitoring allowed us to reconstruct the recovery and range expansion of wolves in an intensively used cultural landscape. By constructing a detailed pedigree, we assessed dispersal distances, pack dynamics and trends in genetic diversity during the early phase of the recolonization process.

### Founder effect and recolonization process

Various studies on wolves in Europe and North America have suggested that natural wolf colonization is usually characterized by frequent long-distance dispersal events and the occurrence of several founders (Mech and Boitani 2003; Fabbri et al. 2007; Åkesson et al. 2016; Ražen et al. 2016). Previous genetic analyses have shown that the first wolves recolonizing Germany originated from the Baltic wolf population in North-Eastern Poland (Czarnomska et al. 2013). Our data suggest that genetic diversity of wolves in Germany has been lost by an initial founder effect at the beginning of the recolonization process, which is consistent with previous population genetic studies on wolf recolonizations in Europe (Fabbri et al. 2007; Granroth-Wilding et al. 2017; Szewczyk et al. 2019). Overall, wolf recolonization in Germany can be described as a rapidly ongoing natural expansion, starting from the initial core area in Eastern Germany close to the Polish border with subsequent colonization in a north-westerly direction (Reinhardt et al. 2019). Wolf expansion in Germany showed comparable patterns to the recovery in Western Poland with subsequent wolf colonization in a north-easterly direction (Nowak and Mysłajek 2016). The haplotype frequencies in our study region are highly similar to that in Western Poland (Czarnomska et al. 2013; Hulva et al. 2018; Szewczyk et al. 2019), underlining that wolves in Germany and Western Poland together form the Central European population (Szewczyk et al. 2019). Besides Poland, colonization patterns in this study resemble respective processes in North America, Scandinavia or in the Alps (Mech and Boitani 2003; Fabbri et al. 2007). Such recolonization processes are characterized by jump expansions in the initial phase, allowing wolves to form packs in areas far from their source populations. When packs establish in new areas and become a source of dispersers, the expansion pattern changes to stratified dispersal, characterised by a combination of long- and short-distance dispersal.

Rapid range expansions may affect spatial genetic patterns of populations (Excoffier et al. 2009; Petit 2011). The recolonization of wolves in Germany was supported by active military training areas that served as stepping-stones and allowed the subsequent formation of wolf packs in the surrounding areas (Reinhardt et al. 2019). The breeders in AG formed the most successful breeding pair during the initial recolonization in Germany, with a large number of successfully reproducing offspring. Analysis of genetic substructure within the population revealed the strong genetic contribution of this pack and its descendants (see Fig. S5). Individuals that were born in the initial core area and their descendants as well as immigrant wolves with an unknown source pack and their descendants also had a strong genetic contribution within the population, providing evidence for allele surfing during range expansion (Excoffier et al. 2009).

### Breeder dispersal and pack dynamics

The most common method of forming a pair is to disperse and find a breeding partner. Depending on their circumstances, wolves make use of several other breeding practices (‘strategies’) to form a pair. The underlying idea is that after reaching sexual maturity every wolf will aim to breed within a wolf population consisting of territorial social groups (Mech and Boitani 2003). We found that both male and female wolves dispersed to seek out a territory and a breeding partner. Although the wolf with the longest dispersal distance was a female, our data did not provide evidence for a significant difference in dispersal distances between the two sexes among dispersers, which is consistent with previous results on wolves (Gese and Mech 1991; Mech and Boitani 2003; Kojola et al. 2006; Jimenez et al. 2017).

Wolves that disperse and successively pair with different breeding partners in different territories often remain undetected, as radio-collared individuals are usually tracked for limited periods of time (Mech and Boitani 2003). Here, the reconstruction of a multigenerational pedigree allowed us to detect multiple dispersal events of male and female wolves during the first years of recolonization. In contrast to the results of the dispersal distances described above, we found that dispersal among packs was strongly male-biased. Mostly males immigrated into pre-established packs, becoming the new breeding male, while female offspring often established new packs next to their natal pack or the territory was taken over by female offspring of the original breeders. Similar sex-specific dispersal patterns among packs were observed in Yellowstone National Park (vonHoldt et al. 2008) and in Italy (Caniglia et al. 2014).

In certain cases, successful reproduction of new breeders can occur without any dispersal from the natal pack. If, for instance, food supply in a territory is plentiful, a mature daughter may breed in addition to the established breeding female (Mech and Boitani 2003). Although such multiple breeding within a territory has been proven by telemetry (Reinhardt et al. 2014), we did not find evidence for this or for multiple paternities in a single litter shown for Yellowstone wolves (Koch et al. 2019). However, close relatedness within the packs in combination with the generally low allelic diversity and the practised genetic sampling methods may have led to specific local breeding strategies being occasionally overlooked.

### Genetic diversity and inbreeding

Populations consisting of organisms that form social groups cannot be considered as simple randomly mating subpopulations, as social structure and sex-biased dispersal influence genetic diversity (Sugg et al. 1996). The rise of genetic diversity in the first years of recolonization is likely due to the proportionally high immigration rate of males and the fact that wolves live in social groups. Indeed, social structure may effectively enhance genetic diversity and reduce inbreeding (Parreira and Chikhi 2015). Overall, the trends in genetic diversity fit to the pedigree data and the pedigree-based inbreeding coefficients (Fp), indicating immigration of new breeders, high sociality and an increasing number of inbred litters in the later years.

Various studies indicate that breeding with close relatives is rather rare in wolves and wolves are usually able to avoid inbreeding within natal packs (Smith et al. 1997; vonHoldt et al. 2008; Geffen et al. 2011; Caniglia et al. 2014). However, this phenomenon may simply be regarded as the result of breeding competition and territorial social group structure (i.e., a failure of young wolves, which lose in the breeding competition with more mature wolves; Mech and Boitani 2003). Geffen et al. (2011) have suggested that inbreeding avoidance in canids may be lacking outside natal groups, as low kin encounter rate and social organization are sufficient to prevent inbreeding. Our findings also suggest that wolves pair indiscriminately with any potential breeding partner outside their natal pack.

We found several cases of inbreeding between close relatives during wolf expansion in Germany, including full-sibling breeding events outside the natal territories in four of out the five cases. The exceptional case relates to male GW038m, born in the NO pack. GW038m first mated with his female cousin in DN, then with his daughter in DN, followed by the full-sibling breeding event with his sister in NO. His sister reproduced with another male before they mated as full-siblings. GW038m dispersed before his sister and later breeding partner was born. In the other four cases, the full-siblings were born in their natal packs in the same or the following year. Thus, the full-siblings probably grew up together in their natal packs before they dispersed. However, three full-sibling breeding events followed previous pair bonds and two full-sibling breeding events occurred about 180-km far from the natal packs. Interestingly, several of the AG descendants reproduced with close relatives, including three full-sibling breeding events. The offspring of the full-sibling breeding pair in HO in 2015 showed the highest pedigree-based inbreeding coefficient (Fp = 0.379), as their great-great-grandparents were already related. In this context, it should be noted that the family ties of individuals with unknown origin are unresolved in this study. Thus, the degree of inbreeding is likely underestimated throughout the dataset (Robinson et al. 2013; Kardos et al. 2018; Robinson et al. 2019). However, inbreeding coefficients found in this study are far lower than those found in the inbred populations of Scandinavia (Liberg et al. 2005; Åkesson et al. 2016) or the Isle Royale wolves (Hedrick et al. 2014), for instance.

## Conclusions

This study documents the rapid recolonization of wolves in the intensively used cultural landscapes of Central Europe. We observed (i) signs of a founder effect and (ii) a colonization process in Germany similar to that found in other areas. We also detected that (iii) gene flow and dispersal among packs were predominantly male-biased, while average dispersal distances did not differ by sex among dispersers. Furthermore, we found (iv) moderate genetic diversity and inbreeding levels of the recolonizing population compared to other European wolf populations (Hindrikson et al. 2017).

The reconstructed pedigree in this study documents close relationships, including several inbreeding events within the expanding wolf population. High sociality, dispersal among packs and immigration of individuals likely from Poland were common during recolonization and helped foster the observed positive trends of allele numbers and heterozygosity as well as relatively low levels of overall inbreeding. The detected levels of heterozygosity and inbreeding in this study may reflect the species-specific behaviour in a re-expanding population with a limited number of unrelated breeding partners and cryptic population structure resulting from strong allele surfing effects in the newly recolonized areas.

The annual population growth rate of wolves in Germany of about 36% (Reinhardt et al. 2019) is higher than detected in the recolonizing Scandinavian wolf population (29%; Wabakken et al. 2001) and similar to the rate of increase of the Central European population in Western Poland (38%; Nowak and Mysłajek 2016).

This rapid return of the wolf into its historic ranges occurred within several human-dominated landscapes in Germany, comprising intensively managed forests, large agricultural areas, dense traffic networks and many urban areas. Although anthropogenic mortality (traffic or poaching) is high (Reinhardt et al. 2019), the presence of large areas of still unoccupied suitable habitat (Kramer-Schadt et al. 2020) with high densities of wild ungulates in combination with the strict legal protection provides favourable conditions for a further expansion of the wolf population. We expect that, in the near future, gene flow between the Central European population and adjacent populations will increase with continuing expansion, resulting in higher genetic diversity.

## Supplementary information

Supplemental Material Figures S1-S5

Supplemental Material Tables S1-S5

## Data Availability

Wolf microsatellite genotype data available from the Dryad Digital Repository: 10.5061/dryad.brv15dv90.
