# Supplementary material for: How the west was won: genetic reconstruction of rapid wolf recolonization into Germany’s anthropogenic landscapes
Source: Heredity (Edinb). 2021 Apr 12;127(1):92–106. doi: 10.1038/s41437-021-00429-6 (PMC8249462; doi:10.1038/s41437-021-00429-6)
Supplement: Supplementary file 1 — Supplemental Material Figures S1-S5 [file 41437_2021_429_MOESM1_ESM.docx]

**
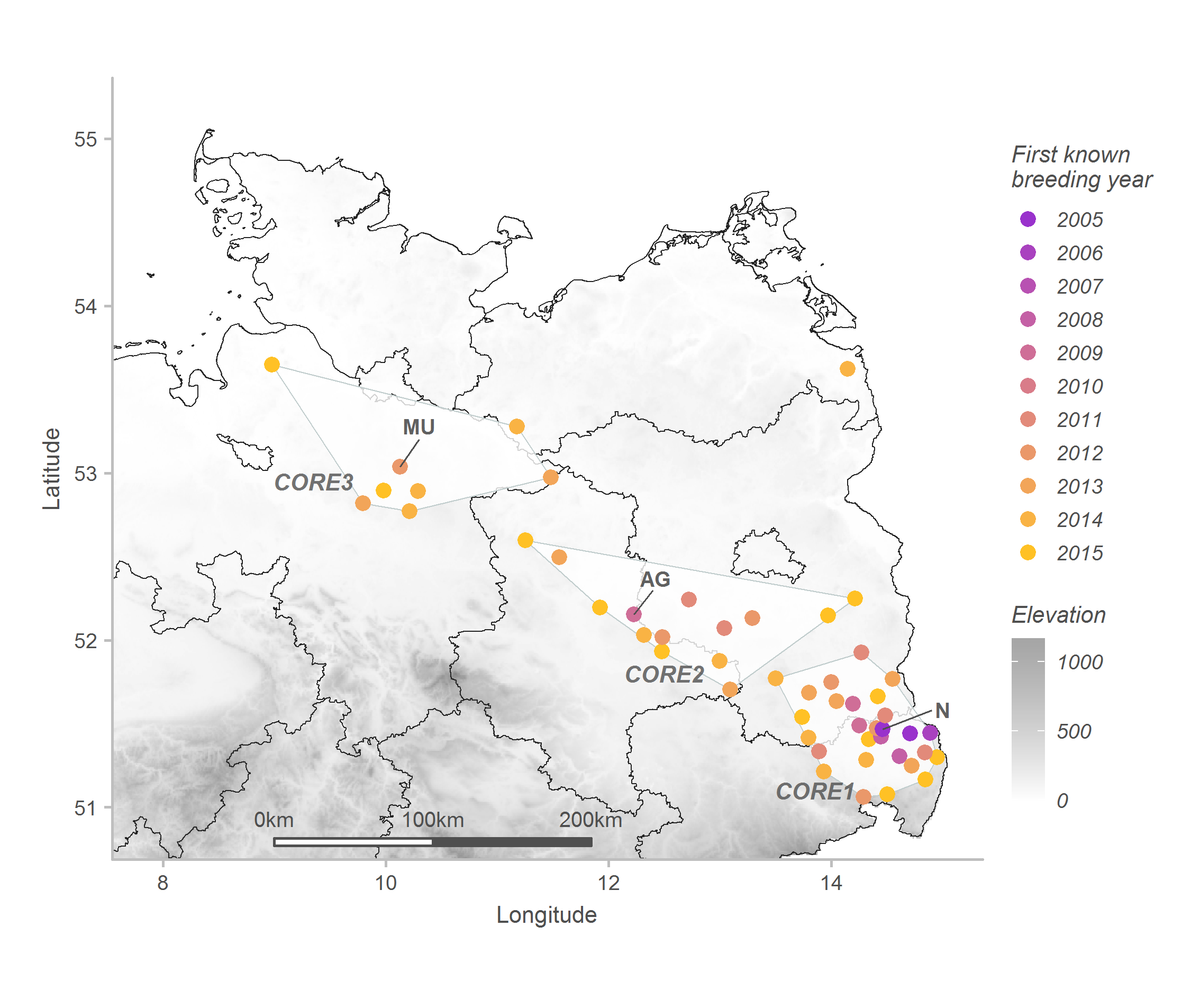
Supplemental Material for Jarausch et al.**

**Fig. S1** Distribution of the three core areas (CORE1, CORE2, CORE3) based on the data of the spatio-temporal expansion process, including territories with genotyped breeding pairs that were identified between 2005 and 2015 in Germany. Colours indicate the first breeding year documented for the respective territory. Labelled are the territories with the first reproduction within the core areas 1 - 3 (N = Neustadt in 2005; AG = Altengrabow in 2009; MU = Munster in 2012). Federal states (grey lines) and elevation (grey shaded)

**
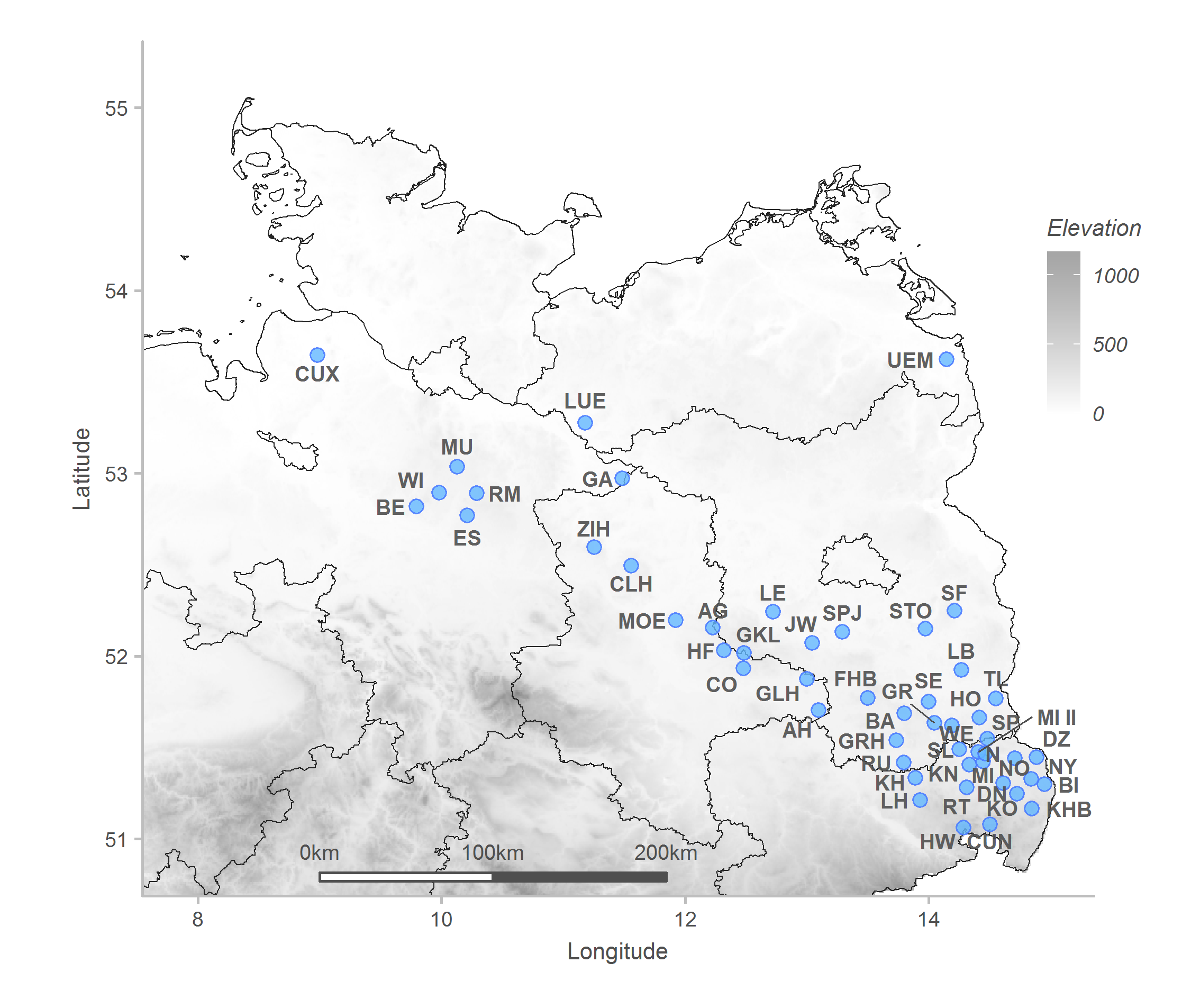
Fig. S2** Distribution of all territories of the genotyped breeding pairs identified between 2005 and 2015 in Germany (blue circles labelled with grey initials, see Suppl. Table S2). Federal states (grey lines) and elevation (grey shaded)


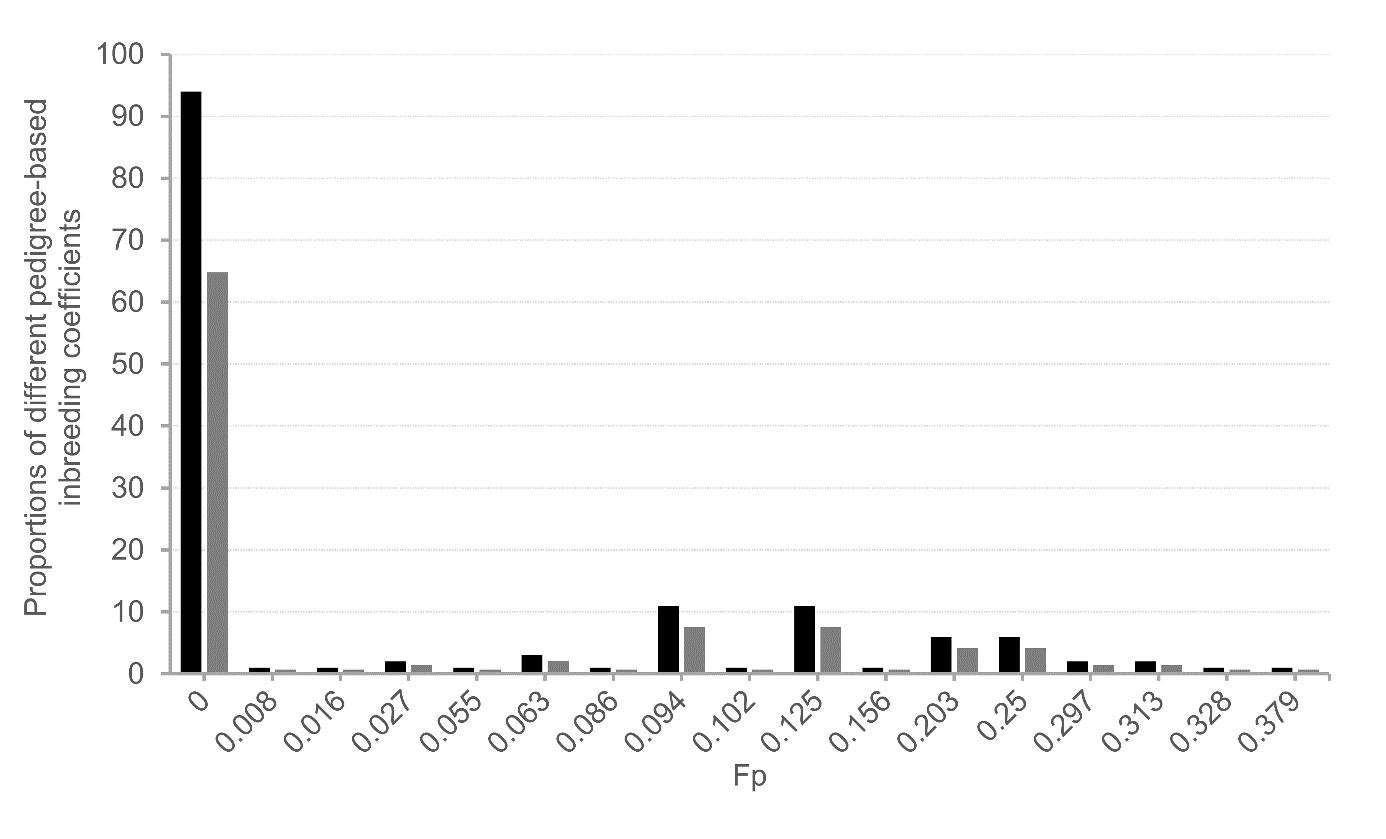
**Fig. S3** Proportions of different pedigree-based inbreeding coefficients (Fp) of the offspring from the genotyped breeding pairs between 2005 and 2015. Proportions of different inbreeding coefficients of the total 145 litters as concrete numbers (black bars) and as percentages (grey bars).


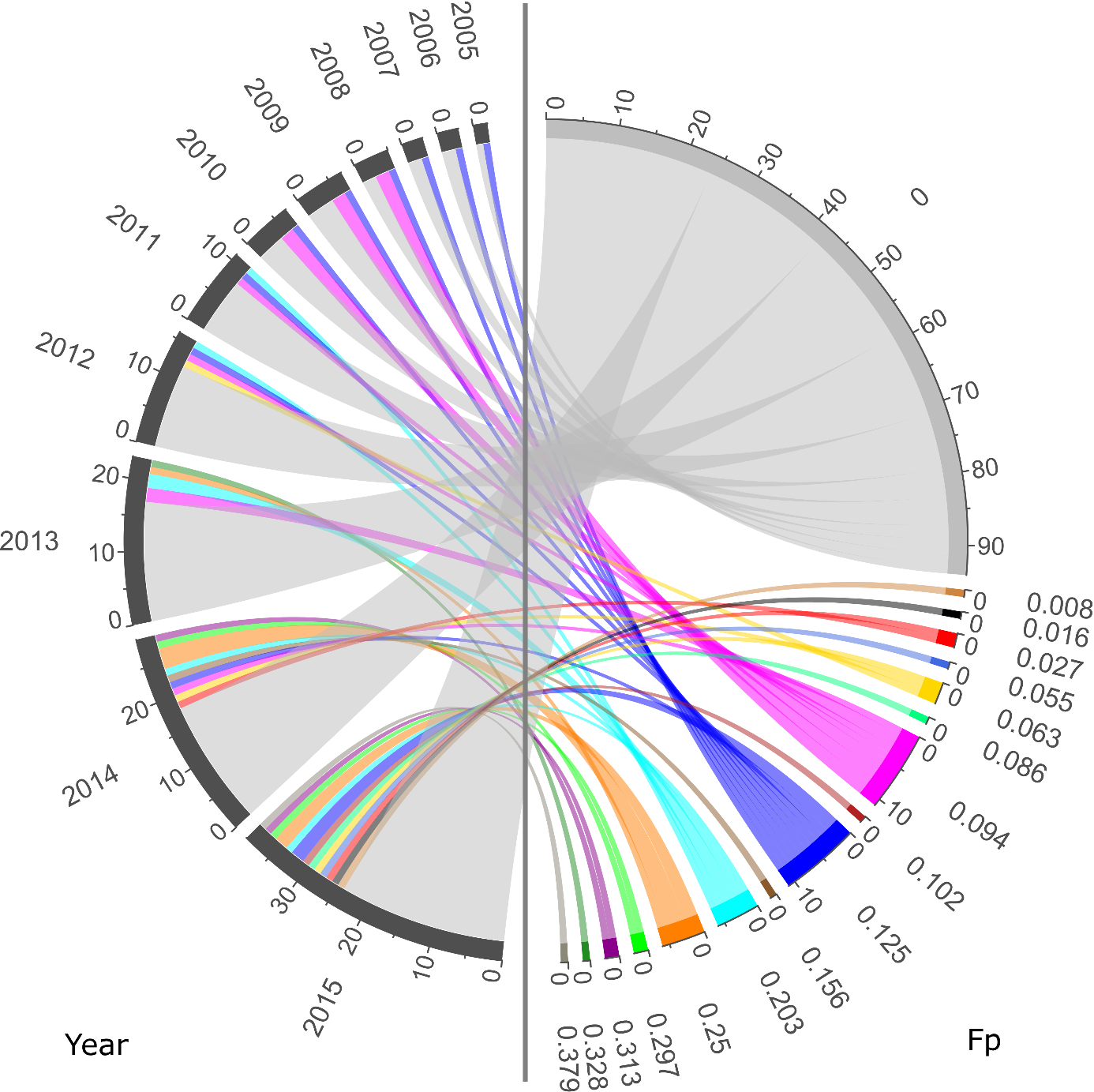


**Fig. S4** Proportions of different pedigree-based inbreeding coefficients (Fp) of the offspring from the genotyped breeding pairs for the years 2005 to 2015. Proportions of different inbreeding coefficients of the total 145 litters as concrete numbers.

**
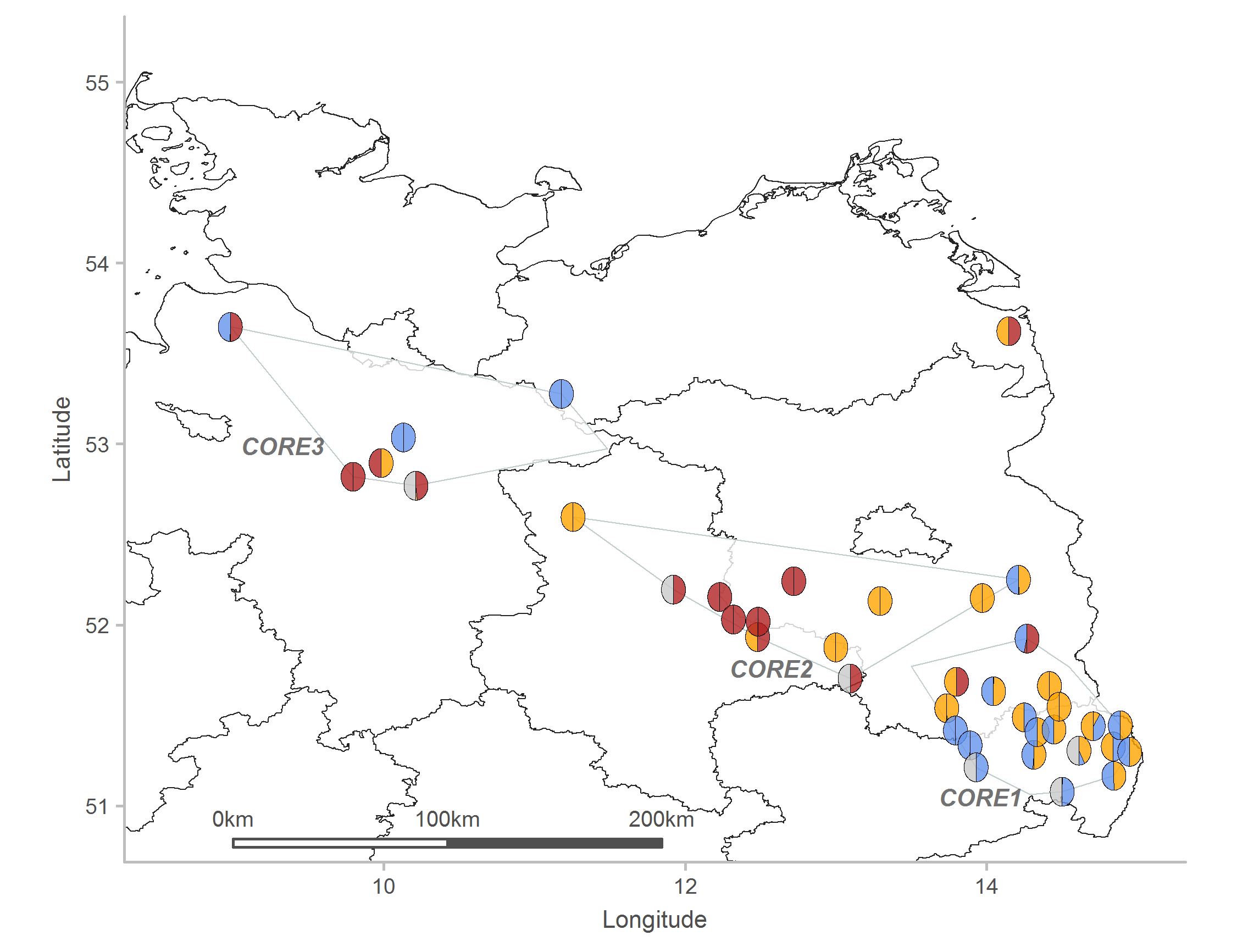
Fig. S5** Distribution of reproducing individuals in the year 2015 for the three core areas that have formed during recolonization and maximum-likelihood clustering results of individual genotypes at K = 3, computed using snapclust. Each pie chart depicts the two breeders (left part = male, right part = female; or left part = breeder with missing genotype (grey segments), right part = genotyped breeder) of the respective territory divided by the vertical line into K colour segments. Proportions of colour segments are equivalent to snapclust individual group membership probabilities. The three clusters represent individuals born in the initial core area (Lausitz) and their descendants (orange), immigrant wolves with unknown source pack and their descendants (blue), as well as the Altengrabow pack and their descendants (dark red).
